# Emergence of *Neisseria gonorrhoeae* Clone with Reduced Susceptibility to Sitafloxacin in China: An In Vitro and Genomic Study

**DOI:** 10.3390/antibiotics13050468

**Published:** 2024-05-20

**Authors:** Meiping Ye, Linxin Yao, Xinying Lu, Fangyuan Ding, Danyang Zou, Tingli Tian, Yi Lin, Zhen Ning, Jianping Jiang, Pingyu Zhou

**Affiliations:** 1 Department of Dermatology, Xinhua Hospital, Shanghai Jiaotong University School of Medicine, Shanghai 200092, China; 2 STD Institute, Shanghai Skin Disease Hospital, Tongji University School of Medicine, Shanghai 200443, China; 3 Division of Tuberculosis and HIV/AIDS Prevention, Shanghai Municipal Center for Disease Control and Prevention, Shanghai 200051, China; 4 Institute of Antibiotics, Huashan Hospital, Fudan University, Shanghai 200040, China

**Keywords:** *Neisseria gonorrhoeae*, sitafloxacin, reduced susceptibility, ST8123

## Abstract

Drug-resistant *Neisseria gonorrhoeae* poses an urgent threat to public health. Recently, sitafloxacin, a new-generation fluoroquinolone, has shown high in vitro activity against drug-resistant *N. gonorrhoeae*. However, data on its effectiveness in clinical isolates remains limited. In this study, we collected 507 *N. gonorrhoeae* isolates from 21 hospitals in Shanghai, China, during 2020 and 2021. Antimicrobial susceptibility testing revealed that sitafloxacin minimum inhibitory concentrations (MICs) exhibited a bimodal distribution, ranging from <0.004 to 2 mg/L. The MIC_50_ and MIC_90_ for sitafloxacin were 0.125 mg/L and 0.5 mg/L, respectively, which are 32 and 16 times lower than those for ciprofloxacin (4 mg/L and 8 mg/L, respectively). Sitafloxacin demonstrated high in vitro activity against isolates resistant to either ceftriaxone, azithromycin, or both. Notably, among the isolates with reduced sitafloxacin susceptibility (MIC ≥ MIC_90_), 83.7% (36/43) were identified as sequence type (ST) 8123. Further phylogenetic analysis showed that ST8123 has evolved into two subclades, designated as subclade-I and subclade-II. A majority of the isolates (80%, 36/45) within subclade-I exhibited reduced susceptibility to sitafloxacin. In contrast, all isolates from subclade-II were found to be susceptible to sitafloxacin. Subsequent genomic investigations revealed that the GyrA-S91F, D95Y, and ParC-S87N mutations, which were exclusively found in ST8123 subclade-I, might be linked to reduced sitafloxacin susceptibility. Our study reveals that sitafloxacin is a promising antibiotic for combating drug-resistant *N. gonorrhoeae*. However, caution is advised in the clinical application of sitafloxacin for treating *N. gonorrhoeae* infections due to the emergence of a clone exhibiting reduced susceptibility.

## 1. Introduction

Gonorrhea remains a major public health challenge, with an estimated 82 million cases reported worldwide in 2020 among adults [1]. The rapid emergence and spread of drug-resistant *Neisseria gonorrhoeae* have exacerbated the situation, prompting the WHO to classify drug-resistant *N. gonorrhoeae* as a high-priority pathogen [2]. Currently, ceftriaxone is the last-resort antibiotic for empirical gonorrhea treatment in most countries, with dual therapy including ceftriaxone and azithromycin recommended for co-infections with pathogens like *Chlamydia trachomatis* and *Ureaplasma urealyticum* [3,4]. A global study of antimicrobial resistance in *N. gonorrhoeae* collected in 2017–2018 found that azithromycin resistance was increasing, and resistance to ceftriaxone and cefixime continued to emerge in multiple counties [5]. A recent study showed that the prevalence of ceftriaxone-resistant *N. gonorrhoeae* approximately tripled in China during 2017−2022 [6]. The rising incidence of resistance to ceftriaxone and azithromycin [7,8] necessitates the exploration of new drugs and treatment strategies to combat drug-resistant *N. gonorrhoeae*.

Sitafloxacin, a new-generation fluoroquinolone antibiotic [9], displays broad-spectrum in vitro activity against various bacteria, such as *Helicobacter pylori* [10], *Mycoplasma genitalium* [11], and even methicillin-resistant *Staphylococcus aureus* [12]. It displayed significant potential for treating ceftriaxone-resistant *N. gonorrhoeae* [13]. Differing from ciprofloxacin and ofloxacin, sitafloxacin demonstrates lower susceptibility to mutations in the quinolone resistance-determining region (QRDR) of *gyrA* (DNA gyrase) and *parC* (topoisomerase IV) [14]. Recent research has highlighted sitafloxacin’s high in vitro effectiveness against ciprofloxacin-resistant *N. gonorrhoeae* strains, particularly those with QRDR mutations, underscoring its potential in treating drug-resistant *N. gonorrhoeae* infections [15,16]. Therefore, comprehensive in vitro studies on a larger scale are essential to further assess sitafloxacin’s efficacy against *N. gonorrhoeae* clinical isolates.

Earlier investigations have shown that mutations in the *gyrA* and *parC* genes, specifically, GyrA-S91F, D95G/A/N, and ParC-S87R/N, D86N, S88P, may contribute to the reduced susceptibility of sitafloxacin; however, all these mutations only increase the MICs of sitafloxacin in *N. gonorrhoeae* to less than 0.5 mg/L [15,16]. Our understanding of *gyrA* and *parC* mutations that confer sitafloxacin resistance is still limited. Furthermore, the knowledge of sitafloxacin-resistant clones is also lacking.

In this study, we collected 507 *N. gonorrhoeae* isolates from 21 hospitals across different regions of Shanghai, China, during 2020 and 2021. Our genomic and phylogenomic analyses identified a clone with reduced susceptibility to sitafloxacin, belonging to the multi-locus sequence type (ST) 8123, within this collection. This reduced susceptibility was potentially determined by the GyrA-S91F, D95Y, and ParC-S87N mutations. Further analysis, incorporating public genome data, revealed that ST8123 strains harboring the GyrA-S91F, D95Y, and ParC-S87N mutations had already appeared in Vietnam in 2012, China in 2013, and the UK in 2014. These findings highlight the early dissemination of a sitafloxacin susceptibility reduced *N. gonorrhoeae* clone prior to the widespread adoption of sitafloxacin, underscoring the urgent need for continuous surveillance of these *N. gonorrhoeae* strains in clinical settings.

## 2. Results

### 2.1. The MIC Distributions of Sitafloxacin and Ciprofloxacin in 507 N. gonorrhoeae Clinical Isolates

**Figure 1:**
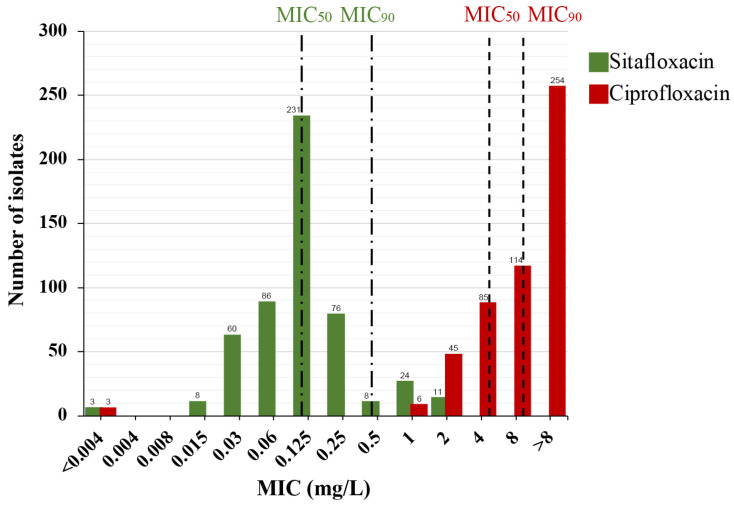
MIC distribution of sitafloxacin (green) and ciprofloxacin (red) against 507 *N. gonorrhoeae* isolates. MIC_50_, MIC where 50% of isolates inhibited. MIC_90_, MIC where 90% of isolates inhibited. MIC_50_ and MIC_90_ of each drug were marked by the dashed lines. The number of isolates is indicated on the top of the bars.

The MICs for ciprofloxacin against the 507 *N. gonorrhoeae* clinical isolates varied widely, from <0.004 to >8 mg/L, with a resistance rate of 99.4% (504/507). In contrast, sitafloxacin MICs ranged from <0.004 to 2 mg/L, with MIC_50_ and MIC_90_ values of 0.125 mg/L and 0.5 mg/L, respectively. These values are 32- and 16-fold lower than those for ciprofloxacin (4 mg/L and 8 mg/L, respectively), suggesting that sitafloxacin exhibits superior in vitro activity against *N. gonorrhoeae*. A total of 43 isolates had sitafloxacin MIC values equal to or greater than MIC_90_ and were defined as sitafloxacin susceptibility reduced strains. A notable bimodal distribution was observed in the sitafloxacin MICs, with peaks at 0.125 mg/L and 1 mg/L (Figure 1), indicating a subpopulation with reduced susceptibility to sitafloxacin within our collection. Given that ceftriaxone and azithromycin are the primary antibiotics for *N. gonorrhoeae* treatment, we also assessed sitafloxacin’s effectiveness against isolates resistant to ceftriaxone (*n* = 57), azithromycin (*n* = 95), and both (dual-resistant) (*n* = 11). The MIC_50_/MIC_90_ values of sitafloxacin against these resistant isolates were 0.125/0.5 mg/L, 0.125/0.25 mg/L, and 0.125/0.25 mg/L, respectively, mirroring those of the overall population. Nonetheless, the sitafloxacin MICs for these resistant isolates were higher compared to that of the population (Table 1), suggesting potential cross-resistance to sitafloxacin, ceftriaxone, and azithromycin.

### 2.2. N. gonorrhoeae Strains with Reduced Sitafloxacin Susceptibility Were Isolated from Multiple Regions in Shanghai

**Figure 2:**
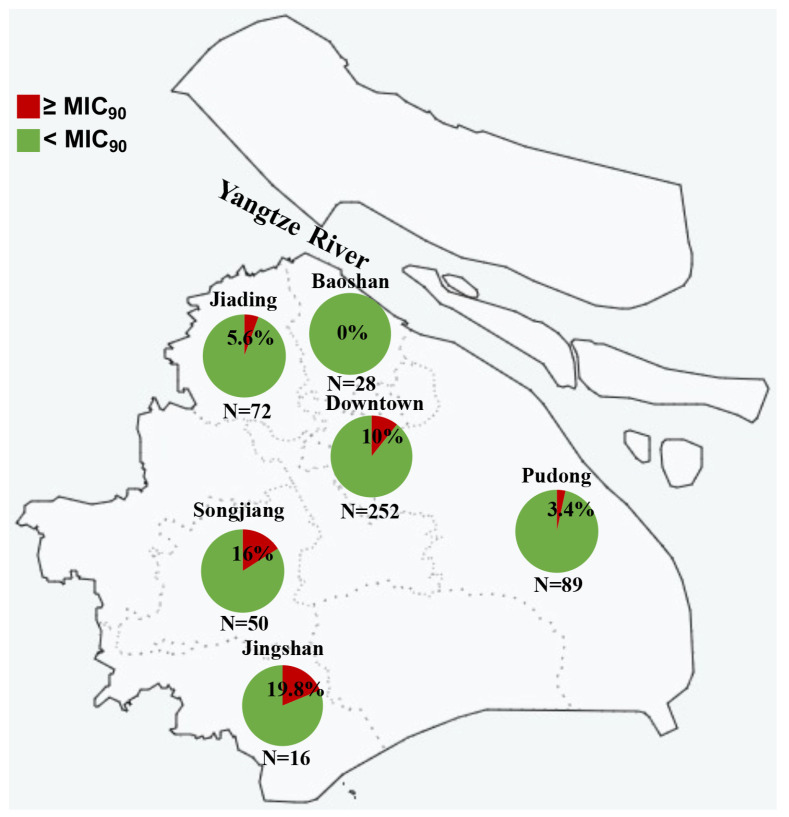
Sitafloxacin MICs of 507 *N. gonorrhoeae* isolated from 6 different regions in Shanghai, China.

To explore the geographical spread of *N. gonorrhoeae* strains with reduced susceptibility to sitafloxacin, we analyzed the 507 strains based on their isolation locations. Figure 2 illustrates that strains exhibiting reduced susceptibility were found across various regions, with rates fluctuating between 0% and 19.8%. Notably, no susceptibility reduced strain was detected in Baoshan, the northern region of Shanghai. Meanwhile, Jiading, another northern area, had four such strains. A significant proportion of strains with reduced susceptibility were identified in the southern regions (Songjiang [*n* = 8], Jingshan [*n* = 3], and Pudong [*n* = 3]) and the downtown area (*n* = 25). This indicates that sitafloxacin susceptibility reduced *N. gonorrhoeae* strains are widely disseminated across Shanghai.

### 2.3. Most of the N. gonorrhoeae Strains with Reduced Susceptibility to Sitafloxacin Belonged to the ST8123 Clone

**Figure 3:**
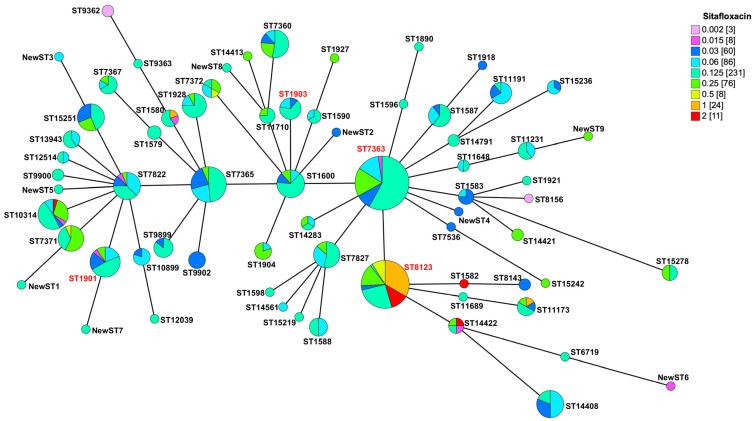
Minimum spanning tree of 507 *N. gonorrhoeae* based on the allelic profiles of the 68 MLST STs isolated. The ST labels in red are major clones of *N. gonorrhoeae*.

In our study, we sequenced all 507 *N. gonorrhoeae* isolates and constructed a minimum spanning tree (MST) based on the multi-locus sequence typing (MLST) (Figure 3). This MST underscored the genetic diversity within our collection, identifying 59 known sequence types (STs) and nine new STs. Predominant among these were ST7363 (*n* = 88) and ST8123 (*n* = 66), together representing 30.4% of all isolates. We also identified ST1901 (*n* = 21) and ST1903 (*n* = 9), known globally for their resistance to cefixime and ceftriaxone. Notably, a significant majority of the isolates with reduced susceptibility to sitafloxacin (83.7%, 36/43) were classified as ST8123, which exhibited MIC_50_/MIC_90_ values of 0.5/2 mg/L, significantly higher than those found in ST7363, ST1901, and ST1903 (Table 2). The presence of reduced susceptibility in other minor STs, such as ST1580, ST1582, and ST14422, was also noted. The pronounced prevalence of reduced sitafloxacin susceptibility within ST8123 suggests a potential underlying mechanism contributing to this phenomenon.

### 2.4. GyrA-S91F, D95Y, and ParC-S87N Mutations May Be Associated with the Reduced Sitafloxacin Susceptibility in N. gonorrhoeae

**Figure 4:**
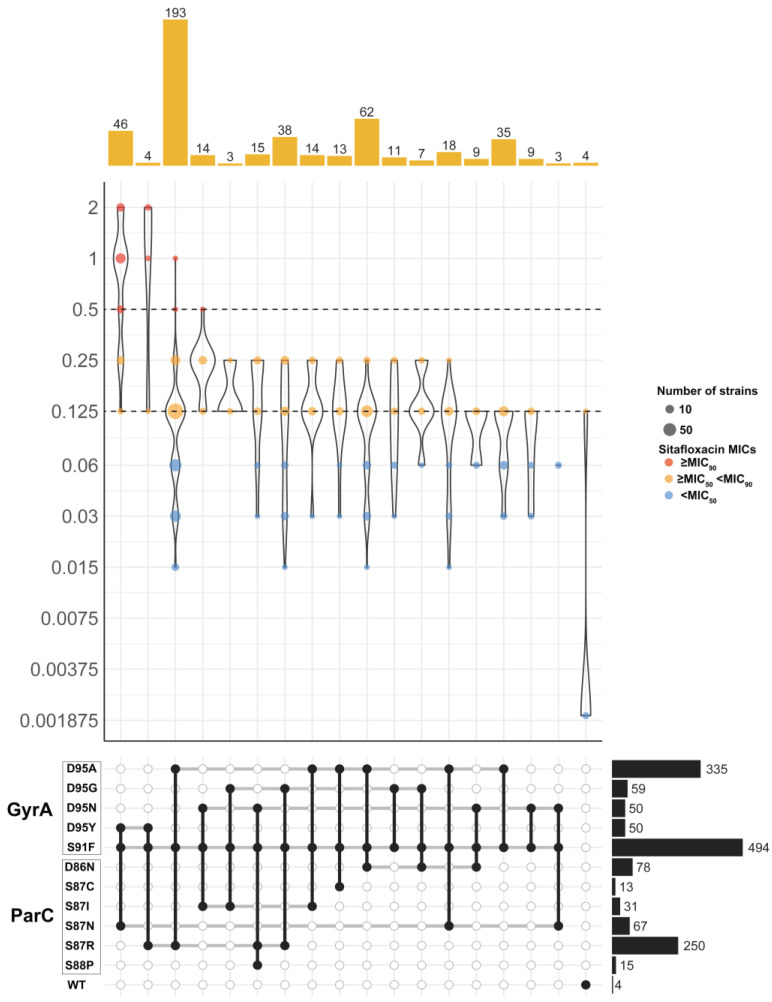
Distribution of MIC values of sitafloxacin in 507 *N. gonorrhoeae* isolates with different combinations of GyrA and ParC mutations. Only combinations observed in at least 5 isolates are shown. Dashed horizontal lines on the violin plots mark the MIC50 and MIC90 for sitafloxacin. Colored dots inside violins represent different MIC ranges. Barplots on the top show the number of isolates with each mutation combination. Combinations of GyrA and ParC mutations were indicated in the grid below by black circles connected vertically. Horizontal thick grey lines connect combinations of mechanisms that share an individual determinant. Barplots on the right show the number of isolates with each mutation.

Investigating the impact of *gyrA* and *parC* mutations on sitafloxacin susceptibility, we found that isolates with these mutations demonstrated a higher sitafloxacin MIC_50_ (0.25 mg/L) compared to wild-type isolates (0.001875 mg/L), indicating that *gyrA* and *parC* mutations negatively affect sitafloxacin’s efficacy (Figure 4). Among these isolates, the common QRDR mutations were GyrA-S91F (99.2%, 503/507), GyrA-D95A (66.1%, 335/507), and ParC-S87R (49.3%, 250/507). We identified 17 significant GyrA and ParC genotype combinations, with groups of less than three isolates excluded from the analysis. Notably, the combination of GyrA-S91F, D95A, and ParC-S87R found in 38% of strains (193/507), exhibited a sitafloxacin MIC range of 0.015 to 1 mg/L (MIC_50_/MIC_90_: 0.125 and 0.25 mg/L). Similarly, GyrA-S91F, D95A, and ParC-S86N were present in 12.2% of strains (62/507), showing the same MIC range. A distinct subset of 46 strains (9.1%) had the GyrA-S91F, D95Y, and ParC-S87N mutations, with a sitafloxacin MIC range of 0.125 to 2 mg/L (MIC_50_/MIC_90_: 1 and 2 mg/L), pointing to a significant reduction in susceptibility. Remarkably, the majority of strains with reduced sitafloxacin susceptibility (83.7%, 36/43) carried the GyrA-S91F, D95Y, and ParC-S87N mutations, and nearly all strains with these mutations (95.7%, 44/46) were classified as ST8123. This suggests that the GyrA-S91F, D95Y, and ParC-S87N mutations are closely associated with reduced sitafloxacin susceptibility.

### 2.5. N. gonorrhoeae Strains with GyrA-S91F, D95Y, and ParC-S87N Were Converged into a Subclade in ST8123

**Figure 5:**
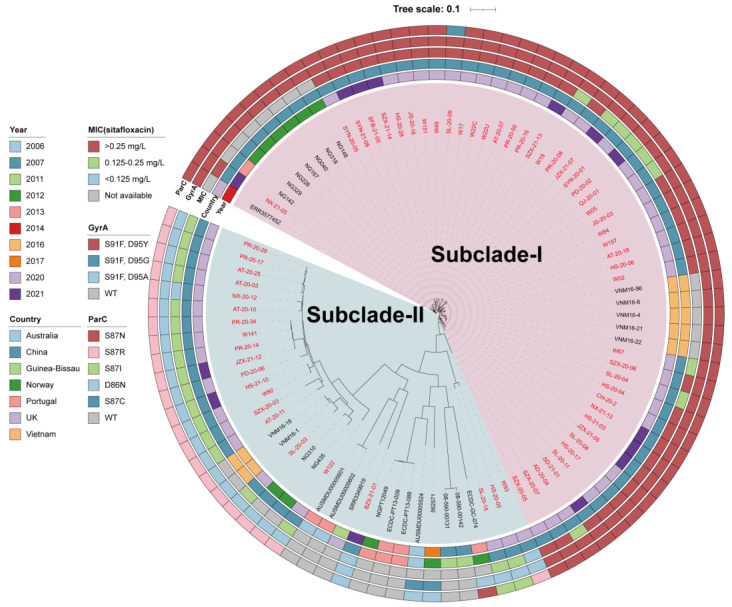
Phylogenic analysis of globally distributed 94 ST8123 *N. gonorrhoeae* strains. Bars from the inner to outer are isolation years, countries, MIC group of sitafloxacin, GyrA mutations, and ParC mutations, respectively. Names in red are isolates sequenced in this study (*n* = 66), while others are from public databases (*n* = 28).

To deepen our understanding of ST8123 *N. gonorrhoeae* strains, we carried out a phylogenomic analysis on 94 ST8123 strains, comprising 66 genomes from our collection and 28 retrieved from public databases. Figure 5 delineates the division of the 94 ST8123 strains into two distinct subclades: subclade-I (*n* = 58), which primarily includes strains from China (*n* = 52) and Vietnam (*n* = 5), with the earliest strain traced back to China in 2012; and subclade-II (*n* = 36), characterized by a more global distribution with strains from China, Vietnam, Portugal, Australia, Norway, Guinea-Bissau, and the UK, the earliest of which was identified in Australia in 2006.

Significantly, subclade-I was marked by a higher incidence of reduced sitafloxacin susceptibility. About 80% of the isolates (36/45) presented MICs > 0.25 mg/L, and 20% (9/45) had MICs between 0.125 mg/L and 0.25 mg/L, excluding strains without MIC data (*n* = 13). Conversely, in subclade-II, 85.7% of strains (18/21) had MICs within the 0.125 to 0.25 mg/L range, and 14.3% (3/21) exhibited MICs <0.125 mg/L, excluding strains without MIC data (*n* = 15). It is noteworthy that all subclade-I strains harbored the GyrA-S91F, D95Y, and ParC-S87N mutations, indicative of reduced susceptibility, except for one with a ParC-S87C mutation. On the other hand, subclade-II predominantly comprised strains with the GyrA-S91F, D95A, and ParC-S87R mutations or wild-type *gyrA* and *parC* sequences.

Furthermore, the strains within subclade-I were genetically closer to each other, suggesting that this group, characterized by reduced sitafloxacin susceptibility, has recently emerged. These findings collectively underscore that the spread and prevalence of ST8123 subclade-I have significantly contributed to the development of sitafloxacin susceptibility reduced *N. gonorrhoeae* in the population.

## 3. Discussion

The rising prevalence of drug-resistant *N. gonorrhoeae* significantly complicates gonorrhea management and narrows the therapeutic options available [17]. Sitafloxacin, known for its broad antibacterial spectrum, emerges as a promising treatment alternative. As of 2017, sitafloxacin has been recommended in Japan as a primary treatment for chlamydial urethritis and a secondary option for non-chlamydial, non-gonococcal urethritis [18]. Moreover, since 2019, China has approved sitafloxacin for treating urinary tract infections and pneumonia when caused by susceptible strains [19]. Recent studies, including ours, have confirmed sitafloxacin’s high in vitro effectiveness against drug-resistant strains of *N. gonorrhoeae*. Our analysis across 507 *N. gonorrhoeae* isolates from Shanghai, China, has pinpointed a sitafloxacin susceptibility reduced clone, already spreading across multiple countries before sitafloxacin became widely used. This finding stresses the critical need for vigilant surveillance of sitafloxacin susceptibility in clinical settings.

In our investigation, the MIC_50_ and MIC_90_ values of sitafloxacin for *N. gonorrhoeae,* estimated at 0.125 mg/L and 0.5 mg/L, respectively, are consistent with reports from Japan and Sweden [16,20], confirming sitafloxacin’s potent in vitro activity against *N. gonorrhoeae* within China as well. Differently from other studies, however, our research revealed a bimodal distribution in sitafloxacin MICs, signifying a notable number of isolates with MICs exceeding the MIC_90_. This suggests the existence of a subpopulation with reduced susceptibility to sitafloxacin. Our subsequent phylogenomic analysis accurately identified this subpopulation as belonging to a specific subclade of ST8123 *N. gonorrhoeae*, which is prevalent across Shanghai. Notably, although reports of ST8123 on an international scale are rare, it is the most common sequence type in Shenzhen, a major southern coastal city in China, from 2014 to 2018 [21]. This finding implies the spread of a sitafloxacin susceptibility reduced *N. gonorrhoeae* clone within China, especially in its foremost coastal cities.

Earlier investigations have pinpointed mutations in the *gyrA* and *parC* genes, specifically, GyrA-S91F, D95G/A/N, and ParC-S87R/N, D86N, S88P, as primary contributors to the reduced susceptibility of sitafloxacin, albeit maintaining MICs below 0.5 mg/L [15,16]. Consistent with these findings, 89.3% of the isolates in our study harbored these mutations, aligning with the observed MICs under 0.5 mg/L. Notably, our research goes a step further by uncovering a novel mutation combination: GyrA-S91F, D95Y, and ParC-S87N. This combination was associated with reduced susceptibility to sitafloxacin, reflected by MICs exceeding 1 mg/L. This suggests that the GyrA-D95Y mutation might significantly impair sitafloxacin’s binding efficacy, underscoring a potentially critical mechanism for diminished drug effectiveness.

Pharmacokinetic studies have shown that a single oral dose of sitafloxacin, at 100 mg or 200 mg, can attain maximum serum concentrations of 1.62 mg/L and 2.73 mg/L, respectively, with the drug’s mean half-life ranging between 5.80 to 6.28 h [22]. These pharmacokinetic properties suggest that a 200 mg dose of sitafloxacin could maintain serum concentrations above 0.5 mg/L for approximately 12 h. This duration and concentration level could be sufficient to effectively target gonococcal strains exhibiting MICs under 0.5 mg/L. Nonetheless, strains harboring the GyrA-S91F, D95Y, and ParC-S87N mutations, which indicate reduced susceptibility, might necessitate higher or repeated dosing of sitafloxacin for successful eradication.

MLST clones such as ST7363, ST1901, and ST1903, known for their resistance to cefixime and ceftriaxone, have been identified globally [21,23,24,25,26] and linked to treatment failures, particularly concerning dual therapy with ceftriaxone and azithromycin in the UK [27]. In our analysis, these sequence types were present, displaying sitafloxacin MICs within the ranges of 0.015 to 0.25 mg/L for ST7363 and ST1901, and 0.03 to 0.125 mg/L for ST1903, all of which are beneath the achievable serum concentrations post-administration. This positions sitafloxacin as a potentially effective alternative for managing infections resistant to or in cases of allergy to ceftriaxone. Of note, the close phylogenetic relationship between ST7363 and ST8123 raises concerns over the potential development of dual resistance under the selective pressure of widespread sitafloxacin use. Thus, ongoing surveillance for drug-resistant *N. gonorrhoeae* remains essential to thwart the rise and dissemination of multi-drug resistant strains.

## 4. Materials and Methods

### 4.1. N. gonorrhoeae Strains Isolation

A total of 507 *N. gonorrhoeae* isolates were consecutively collected from patients who attended STD clinics and dermatology departments across eight different regions of Shanghai within the Shanghai Gonococcal Resistance Surveillance Programme (SH-GRSP) during 2020 and 2021. Neither the gender nor the patients’ symptomatic state was presented in this study. The isolates were cultured on Thayer–Martin (TM) agar (Comagal, Shanghai, China) and incubated at 37 °C in a humidified atmosphere containing 5% CO_2_. For preservation, the isolates were suspended in tryptic soy broth (BD, Franklin Lakes, NJ, USA) with glycerol and stored at −80 °C until further use.

### 4.2. Antimicrobial Susceptibility Testing

Ciprofloxacin (Sigma, Darmstadt, Germany), sitafloxacin (MeilunBio, Dalian, China), ceftriaxone (China National Institutes for Drug control, Beijing, China), and azithromycin (Shanghai yuanye Bio-Technology Co., Ltd., Shanghai, China) powders were bought from manufacturers. The minimum inhibitory concentrations (MICs) for the isolates against these antibiotics were determined via the agar dilution method, following the Clinical and Laboratory Standards Institute (CLSI) guidelines (www.clsi.org, accessed on 1 July 2023). For each testing run, the *N. gonorrhoeae* reference strain ATCC 49226 served as a quality control. Resistance breakpoints for ciprofloxacin, ceftriaxone, and azithromycin were established at ≥1 mg/L, ≥0.125 mg/L, and ≥1 mg/L, respectively, in line with the WHO Global Gonococcal Antimicrobial Surveillance Programme (GASP). As there is currently no defined resistance breakpoint for sitafloxacin, MICs equal to or greater than the MIC_90_ were considered indicative of reduced susceptibility.

### 4.3. Whole Genome Sequencing and Bioinformatics Analysis

Genome sequencing was conducted in accordance with previously established protocols [28]. Briefly, genomic DNA from *N. gonorrhoeae* isolates was extracted using a commercial bacterial genomic DNA extraction kit and sequenced with the Illumina HiSeq platform, utilizing 150-bp paired-end sequencing technology. The sequencing reads were then assembled using SPAdes V3.8 [29] with the default settings, excluding any contigs shorter than 500 nucleotides. Mutations in *gyrA* and *parC* were identified through pyngSTar [30], employing a database sourced from CARD [31] (https://ngstar.canada.ca, accessed on 1 March 2023). The sequence type was determined using the mlst tool (https://github.com/tseemann/mlst, accessed on 1 March 2023) against the pubMLST profiles. The minimum spanning tree was created by GrapeTree v1.5.0 [32]. Associations between QRDR mutations and MIC values were illustrated using a script from a study by Leonor [33] (https://gitlab.com/cgps/pathogenwatch/publications/-/tree/master/ngonorrhoeae, accessed on 1 March 2023). The genes were annotated by Prokka v1.14.5 [34], and the core genome alignment was generated by Roary v3.13.0 [35]. The core genome phylogenetic tree was constructed via the Neighbor-Joining method using MEGA v11 software [36] and visualized with iTOL v5 [37]. *N. gonorrhoeae* genome sequences were retrieved from the pubMLST database [38] as of December 2022 (Table S1).

### 4.4. Statistical Analysis

Rank Sum Tests in R were used to test the difference of MICs between ceftriaxone-resistant isolates, ceftriaxone-susceptible isolates, azithromycin-resistant isolates, azithromycin-susceptible isolates, ceftriaxone and azithromycin-resistant isolates, and all the isolates. Rank Sum Tests were used to test the difference in MICs between different ST clones. A *p*-value less than 0.05 was considered statistically significant.

## 5. Conclusions

In conclusion, our study contributes significant insights into the effectiveness of sitafloxacin against *N. gonorrhoeae* clinical isolates within China. While sitafloxacin demonstrated high efficacy against the majority of tested isolates, our analysis has unveiled the emergence and spread of a ST8123 subclade exhibiting reduced susceptibility to sitafloxacin. These findings underscore the need for cautious application of sitafloxacin in the clinical treatment of gonorrhea, highlighting the importance of ongoing surveillance of drug-resistant gonococcal strains.

## Figures and Tables

**Table 1:** The sitafloxacin MIC ranges of isolates resistant or susceptible to ceftriaxone or (and) azithromycin.

Antimicrobials (No. of Isolates)	Sitafloxacin MIC (mg/L)			*p* Value (vs. All)
	Range	MIC_50_	MIC_90_	
Ceftriaxone-Resistant isolates (57)	0.03–2	0.125	0.5	0.001 **
Ceftriaxone-Susceptible isolates (450)	<0.004–2	0.125	0.25	0.8
Azithromycin-Resistant isolates (95)	0.03–2	0.125	0.25	<0.001 ***
Azithromycin-Susceptible isolates (412)	<0.004–2	0.125	0.25	0.9
Ceftriaxone and Azithromycin-Resistant isolates (11)	0.125–0.5	0.125	0.25	0.014 *

* *p* < 0.05, ** *p* < 0.01, *** *p* < 0.001.

**Table 2:** The sitafloxacin MIC ranges of isolates with different STs.

MLST (No. of Isolates)	Sitafloxacin MIC (mg/L)			*p* Value	
	Range	MIC_50_	MIC_90_	vs. All	vs. ST8123
ST8123 (66)	0.03–2	0.5	2	<0.001 ***	
ST7363 (88)	0.015–0.25	0.125	0.25	0.69	<0.001 ***
ST1901 (21)	0.015–0.25	0.125	0.25	0.96	<0.001 ***
ST1903 (9)	0.03–0.125	0.125	0.125	0.82	<0.001 ***

*** *p* < 0.001.

## Data Availability

The genome sequences of *N. gonorrhoeae* isolates have been deposited in the DDBJ/ENA/GenBank under the bioproject PRJNA956288.

## Supplementary Materials

The following supporting information can be downloaded at https://www.mdpi.com/article/10.3390/antibiotics13050468/s1, Table S1. The meta data of all the ST8123 strains.
